# Do proton pump inhibitors increase mortality? A systematic review and in‐depth analysis of the evidence

**DOI:** 10.1002/prp2.651

**Published:** 2020-09-30

**Authors:** Mohamed Ben‐Eltriki, Carolyn J. Green, Malcolm Maclure, Vijaya Musini, Ken L. Bassett, James M. Wright

**Affiliations:** ^1^ Therapeutics Initiative Drug Assessment Working Group University of British Columbia Vancouver Canada; ^2^ Department of Anesthesiology, Pharmacology & Therapeutics Faculty of Medicine University of British Columbia Vancouver Canada; ^3^ Department of Family Practice Faculty of Medicine University of British Columbia Vancouver Canada; ^4^ Department of Medicine Faculty of Medicine University of British Columbia Vancouver Canada

**Keywords:** long‐term use, medication harms, mortality, mortality studies, pharmacovigilance, proton pump inhibitors, systematic reviews

## Abstract

Proton pump inhibitors (PPIs) were primarily approved for short‐term use (2 to 8 weeks). However, PPI use continues to expand. Widely believed to be safe, we reviewed emerging evidence on increased mortality with PPI long‐term use. Our 2016 systematic PPI drug class review found that mortality was not reported as an outcome in randomized controlled trials (RCTs) that directly compared different PPIs. We sought more recent and comprehensive data on PPI harm outcomes from research syntheses as a follow‐on. A search was conducted from January 2014 to January 2020. We searched MEDLINE, EMBASE, and Cochrane Central for evidence from systematic reviews (SRs) and primary studies reporting all‐cause mortality in adults treated with a PPI for any indication (duration >12 weeks) compared to patients without PPI treatment (no use, placebo, or H2RA use). Two independent investigators assessed study eligibility, synthesized evidence, and assessed the quality of the included studies. Data on all‐cause mortality were sought, analyzed, critically examined, and interpreted herein. From 1304 articles, one SR was identified that reported on all‐cause mortality. The SRs pooled three observational studies with data to 1 year: odds ratio, 95% confidence interval (CI) 1.53‐1.84. A RCT, the COMPASS (Cardiovascular Outcomes for People Using Anticoagulant Strategies) RCT with data to 3 years: hazard ratio (HR) 1.03, 95% CI 0.92‐1.15. The US Veterans Affairs cohort study using a large national dataset with data to 10 years found a HR of 1.17, 95% CI (1.10‐1.24) and (NNH) of 22. The most common causes of death were from cardiovascular and chronic kidney diseases, with an excess death of 15 and 4 per 1000 patients, respectively, over the 10‐year period. Harms arising from real‐world medication use are best evaluated using a pharmacovigilance “convergence of proof” approach using data from a variety of sources and various study designs. Given that most PPI indications for use recommended a treatment duration of less than 12 weeks, it seems clear that PPIs were significantly overused in older patients. The median exposure time to PPI ranged from 1 to 4.6 years. Signals of serious harms including increased mortality with long‐term PPI use are reported in observational studies. The COMPASS trial findings are not inconsistent with contemporaneous findings from observational studies. The COMPASS RCT was unlikely to detect an increase in mortality given the trial was not powered to detect this outcome. The potential increase in mortality in older patients associated with prolonged PPI exposure needs to be conveyed to health professionals. Clinicians and patients may be able to reverse the relentless expansion of long‐term PPI exposure by reviewing indications and considering potential harms as well as benefits.

**Abbreviations**
ASA: acetylsalicylic acid
C: comparator
CI: confidence interval
COMPASS: Cardiovascular Outcomes for People Using Anticoagulant Strategies
CV: cardiovascular
H2RA: histamin‐2 receptor antagonist
HR: hazard ratio
I: intervention
n: number
NNH: number needed to harm
O: outcome
OR: odds ratio
P: population
PPI: proton pump inhibitor
RCT: randomized controlled trial

## 1 INTRODUCTION

Prescription proton pump inhibitors (PPIs) are primarily approved for short‐term use (2 to 8 weeks) for peptic ulcer disease (PUD), reflux esophagitis, and nonulcer dyspepsia.^1^ However, PPI use continues to expand. In British Columbia, Canada for example, 64% of adults ≥age 65 with a prescription for a PPI in 2018 had a cumulative exposure exceeding 2 years; 44% exceeded 5 years.

Long‐term PPI use is approved by regulators and/or endorsed by gastroenterologists for prevention of gastric damage associated with the adverse effects of other drugs, gastric bleeding, severe esophagitis or Barrett's esophagus, or to prevent gastric damage associated with adverse effects of other drugs, all indications, which only account for a small proportion of long‐term PPI use in Canada.^2^, ^3^ While studies of patient populations with indications for long‐term use are worthy of study, this group is out of scope for our review. Unnecessary overuse has not been identified as a concern in this population.

The short‐term benefits of PPIs as a drug class are not disputed.^2^, ^3^, ^4^, ^5^ However, the belief that the positive net benefit to harm ratio with short‐term treatment extends to long‐term use (greater than 12 weeks) has been challenged by postmarket analyses.^6^, ^7^, ^8^, ^9^

Health Canada^10^ has issued warnings for a number of adverse events and drug interactions that were not recognized when the first PPIs were approved 30 years ago: hypomagnesemia accompanied by hypocalcemia and hypokalemia (2011), *clostridium difficile*‐associated diarrhea (2012), bone fractures (2013), subacute cutaneous lupus erythematosus (2017), as well as new drug interactions with clopidogrel (2009) and methotrexate (2012). There are US Food and Drug Administration warnings for PPI use and risk of increased risk of bone fractures, *clostridium difficult* infection (*CDI*), and profound hypomagnesemia.

A number of professional associations and independent drug bulletins recommend reducing PPI exposure and provide tools for deprescribing.^11^, ^12^ Encouraging restraint has yet to achieve a measurable impact on long‐term PPI prescribing for the common indications. Is the evidence of harms sufficient that we should intensify efforts to constrain new prescriptions and to deprescribe for long‐term users?

In a systematic review conducted by our group in 2016, we reported on the comparative effectiveness of PPIs, benefits, and harms, as well as evidence for considering deprescribing.^2^, ^3^ In many clinical settings, we do not know whether the benefits of long‐term PPI use outweigh the harms. Harms were underreported in RCTs that directly compared different PPIs. Mortality, serious adverse events, and withdrawal due to adverse events were not reported.^2^, ^3^ We found no long‐term, head‐to‐head comparative RCTs that were specifically designed to monitor adverse effects of PPIs.

Recent evidence from a clinical trial^13^ has raised doubts on a growing consensus from observational studies and systematic reviews (SRs) of observational studies that PPI exposure is associated with increased risk of death; the risk increases with increased exposure.^14^, ^15^, ^16^ Therefore, the aim of this review was to summarize and critically examine evidence from SRs and primary studies reporting all‐cause mortality.

## 2 MATERIALS AND METHODS

### 2.1 Searching strategy

Recently in our 2016 systematic review, mortality outcome was not reported in RCTs that directly compared different PPIs.^2^, ^3^ An updated search was performed by information specialist from January 2014‐the date of our last comprehensive search and PPI class review to January 2020 in the following databases: PubMed, MEDLINE, EMBASE (through Ovid), the Cochrane Central Register of Controlled Trials (CENTRAL), and the Cochrane Database of Systematic Reviews. The combination of the following medical subheadings (MeSH) and keywords was used for database searching: proton pump inhibitors or PPI and adverse events or esomeprazole or pantoprazole or omeprazole or rabeprazole or lansoprazole and any indications. Alternative spellings and abbreviations of the above keywords were also considered with no limitation on the language or the publishing date.

### 2.2 Inclusion criteria

Systematic reviews (with or without meta‐analysis) or primary studies were included that met the following criteria: (Cochrane “PICOS” format):

P—adults aged 18 years or older.

I—PPI therapy for any indication for duration of more than 12 weeks.

C—Non‐use or histamine type‐2 receptor antagonist (H2RA) use.

O—All‐cause mortality.

Primary studies were sought and included that had not been available by SR search cutoff dates up to January 2020.

### 2.3 Data extraction and synthesis

Two investigators (MBE and CJG) independently selected eligible systematic review. Disagreement was resolved by discussion with another investigator (VM). Data on all‐cause mortality were sought, synthesized, analyzed, critically examined, and interpreted from SRs and primary studies. We extracted odds ratio (ORs), relative risk (RRs), or hazard ratios (HRs) from the included studies with 95% CI. We did not reanalyze the authors’ original data or conduct new meta‐analyses by combining studies.

### 2.4 Harm outcome hierarchy

The Therapeutics Initiative analyses all available evidence for harms according to a consistent hierarchy of harm outcomes, ranked by clinical importance starting with all‐cause mortality, cause‐specific mortality, total serious adverse events, and other adverse events. For this study we limited our reporting of findings to all‐cause mortality and cause‐specific mortality.

## 3 RESULTS

**Figure 1:**
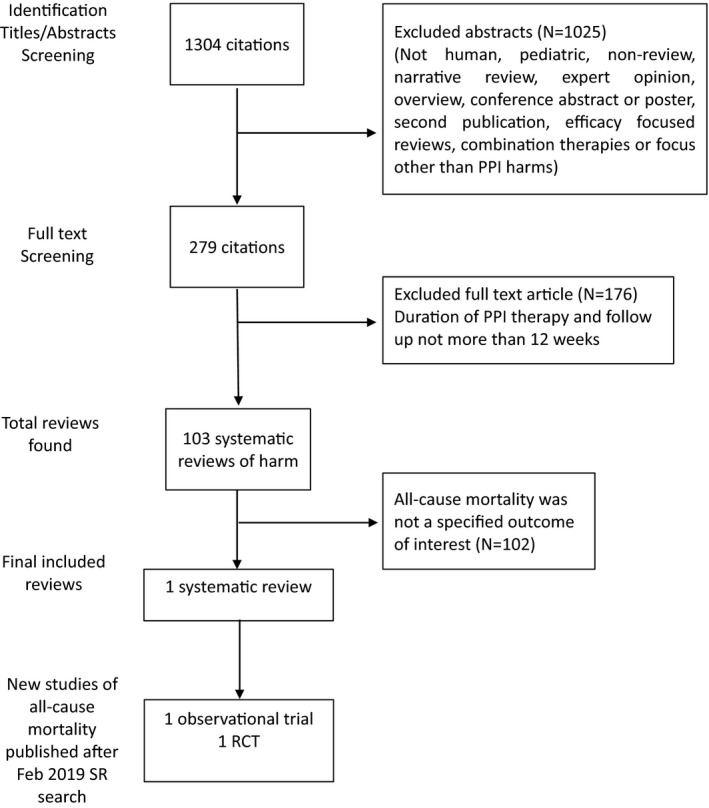
The flowchart of study selection for all‐cause mortality with PPI use

Three recent studies reporting on all‐cause mortality with PPI use were identified that met our inclusion criteria; each having a different study design.^17^, ^18^, ^19^ One systematic review out of 103 was identified that specifically included all‐cause mortality as an outcome in its protocol. A RCT and a longitudinal cohort study that were published after the date of our search for SRs met our inclusion criteria. Figure 1 shows selection process and provides the reasons why some articles were excluded. Table 1 provides detailed characteristics of the included studies.

**Table 1:** Characteristics of included studies

Author Year (Reference)	n. of Patients	Study design n. of studies	PICO	Exposure to PPI	Length of follow‐up (maximum)
Shiraev 2018^17^	22 427	Systemic review of three cohort (prospective and retrospective)	P: elderly > 65 years 90% of patients were on ASA I: PPIs users C: non‐PPIs users O: all‐cause mortality and CV events	Less than 1 year	1 year
Xie 2019^18^	214 467	A longitudinal observational cohort study PPIs (n = 157,625) H2RAs (n = 56,842)	P: elderly > 65 years, men, white I: PPIs user C: H2RAs users O: all‐cause mortality, CV and kidney disease‐specific mortality	4.6 years (median)	10 years
Moayyedi 2019 ^19^	17 598	RCT Harm outcomes were secondary outcomes	P: elderly > 65 years, stable CV disease I: PPIs users C: placebo O: all‐cause mortality	Less than 3 years	3 years

The Shiraev 2018 SRs pooled all‐cause mortality data from three published observational studies.^17^ Eighty‐nine percent of the data was from Charlot et al, 2011, a study of Danish patients following their first myocardial infarction (19,925 of the 22,427 patients in Shiraev 2018).^20^ The pooled mortality rate was higher among PPI users compared with non‐PPI users (OR = 1.68; 95% CI: 1.53 to 1.84; Table 2). In Charlot et al, 2011 mortality was increased during 1‐year follow‐up in people taking PPIs (HR = 2.38; 95% CI: 2.12‐2.67).

**Table 2:** All‐cause mortality estimates during long‐term use of PPI (>12 weeks)

Type of study (Reference)	Deaths n/N (%)	Association (95% Confidence Interval) NNH
Systematic review and meta‐analysis of three observational studies ^17^ Median follow‐up 1 year	PPI: 765/4,775 (16%) Non‐PPI users: 1,794/17,652 (10%)	OR 1.68 (1.53‐1.84)
US Veterans Affairs longitudinal cohort study ^18^ new users of PPI vs. H2RA Median follow‐up 10 years	PPI: 59,771/157,625 (37.9%) H2RA: 20,287/56,842 (35.7%)	HR 1.17 (1.10‐1.24) 45.20 excess deaths/1,000 (28.20‐61.40)
COMPASS RCT ^19^ Pantoprazole 40mg/d vs. placebo Median follow‐up 3 years	PPI: 630/8791 (7.2%) Placebo: 614/8807 (7.0%)	HR 1.03 (0.92‐1.15)

Xie et al, 2019 conducted a longitudinal cohort study emulating a clinical trial using administrative data from the United States’ Veterans Affairs (VA) national database.^18^ New users of acid suppressing medication were identified between July 2002 and June 2004 and followed from their medical records for 10 years. The cohort included 214,467 US veterans (mean age of 65), who newly started taking PPIs (n = 157,625) or H2RAs (n = 56,842). The risk of death was higher with PPI versus H2RA users (HR = 1.17; 95% CI: 1.10 to 1.24). Event rates were 59,771 per 157,625 (37.9%) for PPIs vs 20,287 per 56,842 (35.7%) for H2RAs (Table 2).

A RCT, COMPASS (Cardiovascular Outcomes for People Using Anticoagulant Strategies) Moayyedi et al, 2019, involved a second randomization of participants with heart and peripheral artery disease who were first randomized to rivaroxaban plus ASA or ASA alone. A subgroup without an indication for PPI use or PPI use on entry into the trial was secondarily randomized to receive pantoprazole 40 mg daily vs. placebo. A total of 17,598 participants had no approved indication for PPI treatment; data on adverse events were collected in interviews every 6 months from 580 centers in 33 countries without further verification. The death rates were 630 per 8791 (7.2%) for pantoprazole vs 614 per 8807 (7.0%) for placebo (HR = 1.03; CI: 0.92 to 1.15; (Table 2).^19^

### 3.1 Appraisal of included studies

The included studies used different study designs and can be evaluated using the three sets of quality criteria appropriate for their respective design. Such heterogeneity is appropriate for considerations of medication harm in the real world. Each publication has been peer reviewed and meets sufficient criteria to be valid for the research question, methods and findings presented.

The representativeness across all included studies is problematic as the populations were primarily Caucasian that may limit generalizability to other populations. It is known that up to 20% of Asians (vs 3% Caucasians) have low CYP2C19 enzyme activity and are therefore poor metabolizers of PPIs with a doubling of plasma PPI levels and therefore greater exposure.^17^, ^21^

Each study also has limitations within the respective study design. These are highlighted here.

### 3.2 Observational studies

Common to all the included studies is the challenge of misclassification of drug use. Prescription data may not truly reflect drug consumption. Users may have stopped taking PPIs or H2RAs or started taking PPI as over‐the‐counter medications during the follow‐up period.

Findings for all included studies may be subject to bias by indication if patients who are more ill are more likely to be prescribed PPI therapy. The logic is that people who are prescribed PPIs are sicker and what has caused them to be sick (and then die) is the residual confounder that also caused them to be prescribed a PPI. Healthy populations were not however well represented in the study populations of any of the analyses and each demonstrated that the control population was comparable on comorbidities as well as characteristics such as age and sex.

The pooled analysis SR by Shiraev 2018 included studies if they “examined death or atherosclerotic events (including myocardial infarct, stroke, or peripheral arterial events), and compared a group exposed to PPIs with a control group (not exposed to PPIs), in any group of patients”.^17^ The search cutoff date of October 2016 was not inclusive of more recent studies including the 2019 studies included in this review. The Danish national health set study that dominates the Shiraev 2018 pooled analysis is limited to a study population after a first heart attack.^20^ The advantage of analyses representative of a geographical population being inclusive of all health‐care transactions in a publicly funded health‐care system is the real‐world perspective. No serious limitations were found using the AMSTAR 2 tool for assessing the methodological quality of SRs; however, the lack of a published a priori protocol and reported conflict of interest among authors are noted limitations.

### 3.3 RCT

There are several reasons for cautious interpretation of the COMPASS trial results. Serious harms such as cardiovascular disease, kidney diseases, or development of cancers over relatively long time periods because of the slow onset. The duration of exposure and follow‐up and consistency with the VA cohort means that serious but relatively rare harm may not have been detected. The authors recognized that low event rates for some outcomes limited their ability “to exclude a modest risk increase” from pantoprazole. Of the three included studies, the COMPASS trial was the only one with potential conflict of interest due to funding of the research and investigators. There is also the challenge of consistently detecting adverse events with a multi‐site, multi‐country interview protocol on a 6‐monthly schedule.

The COMPASS trial is also not consistent with other RCTs which show a clear positive reduction of GI complications in patients taking PPI and no clinical effects on cardiovascular events.^5^, ^22^, ^23^, ^24^ Surprisingly, COMPASS found no benefit of using pantoprazole to prevent upper GI bleeding in this population. The COMPASS effectiveness trial in people using antithrombotic drugs (14) have yet to prove that net benefits exceed harms during long‐term use in older people. The data confirmed no benefit of using pantoprazole to prevent upper GI bleeding in the selected population. This raises questions on the role of PPIs in the prevention of bleeding associated with antithrombotic therapy.

### 3.4 Interpretation

The VA cohort study found an excess of deaths in its sample that included 12 times as many participants as the COMPASS RCT and follow‐up that was over three times longer. Furthermore, the Shiraev 2018 SR pooled analysis was heavily weighted by a study using the Danish national level administrative data collected from routine care transactions. It would be difficult to create an RCT of an adverse drug event on the scale of either study.

The median exposure to PPI was longer than in the COMPASS RCT (4.6 years vs <3 years). With only 3 years of follow‐up, COMPASS did not have statistical power to detect 10% increases in risk for several of its prespecified outcomes. For example, COMPASS’s point estimate hazard ratio of 1.17 (0.94 to 1.45) for chronic kidney disease was similar to the VA’s hazard ratio of 1.16 (1.01 to 1.33) for acute kidney injury.

In the COMPASS RCT, pantoprazole increased enteric infections (mostly *C difficile)* with an odds ratio of 1.33 (1.01‐1.75), absolute risk increase of 0.4%. However, the incident rates for most serious harm, such as cardiovascular disease, hospitalizations, chronic kidney disease, or dementia, were consistently higher among pantoprazole users compared to placebo group. The COMPASS authors admit this limitation, yet conclude perhaps inappropriately that PPIs “are not associated with any long‐term harm.” ^13^

The Xie et al, 2019 analysis using VA cohort data went farther than detecting a mortality difference between a new PPI user group and a new H2Ra user group. They traced excess deaths to the underlying cause of death using ICD‐10 (international classification of diseases, 10th revision) codes. Table 3 provides cause‐specific mortality from cardiovascular disease and chronic kidney disease from Xie et al, 2019.^18^ The cardiovascular disease outcome findings from the COMPASS RCT which were available are provided for comparison.^13^ Cause‐specific mortality data are consistent with the overall data analysis as well as consistent with findings of SRs that report on cardiovascular ^17^ and kidney disease.^25^ This consistency is an indication of the VA study's internal validity—the findings are consistent within the study. And the study is consistent with other data^17^, ^25^ which is an indication of external validity—that the findings may be applicable beyond this study population.

**Table 3:** Effect estimates for cause‐specific mortality with PPI use (>12 weeks)

Author, Year (Reference)	Death %	Association (95% Confidence Interval) NNH
Cardiovascular disease		
Shiraev 2018 ^17^	PPI: 2.4% Control: 1.8%	OR 1.54 (1.11‐2.13)
Xie 2019 ^18^	PPI: 8.87% H2RA: 7.33%	HR 1.25 (1.10‐1.44) 15.48 excess deaths/1,000 (5.02‐25.19)
Moayyedi 2019 ^19^	PPI:7.9% Placebo:7.5%	HR 1.04 (0.93‐1.15)
Chronic kidney disease		
Xie 2019 ^18^	PPI: 0.86% H2RA: 0.44%	HR 2.02 (1.31‐3.00) 4.19 excess deaths/1,000 (1.56‐6.58)

There were 17.47 excess deaths from cardiovascular diseases per 1000 patients (95% CI: 5.47‐28.80), NNH of 58, and 6.25 excess deaths from chronic kidney diseases per 1000 patients (95% CI: 3.22‐9.24) in the Xie et al, 2019 study (Table 3) during 10 years of follow‐up.^18^ Moayyedi et al, 2019 did not find an association between PPI therapy and an increased risk of death due cardiovascular causes (HR = 1.04; 95% CI: 0.93 −1.15) compared with placebo; however, there was an overlap in confidence intervals and the COMPASS RCT was shorter in duration and follow‐up.^19^

The Bradford‐Hill criteria provide another framework used to guide an evaluation of the causal association between drugs in the postmarket period and adverse events. Originally developed to examine the causal relationships between public health exposures such as smoking and air pollution (which cannot ethically be randomized) and poor health outcomes it is also a useful framework for evaluating the harm profile of drugs. One of the Bradford‐Hill criteria is biologic plausibility—there is a biological explanation for how the “exposure” could cause the “harm” from what is known.

Xie et al, 2019 report on what may be a universal mechanism of harm with PPI use and one that is consistent with their findings of specific but varied causes of increased mortality. When scientists at the Center for Cardiovascular Regeneration in Huston, Texas, cultured microvascular epithelial cells they aged faster in media with clinically significant amounts of the PPI esomeprazole.^26^ The endothelial cells that line blood and lymph vessels are present throughout the body. Basic science studies showed that exposure to PPIs impaired endothelial lysosomal acidification, enzyme activity, and proteostasis resulting in endothelial dysfunction. Moreover, the telomere length was shortened (a possible sign of aging) in the esomeprazole‐treated group. Xie et al, 2019 also point out that there are two general biological mechanisms by which PPI use can be linked to excess deaths: worsening of preexisting diseases (eg, existing cardiovascular and kidney disease) or the occurrence of new disease states.^18^ This is only one avenue by which long‐term PPI use may adversely affect human health. Also plausible are hypomagnesemia, drug interactions, reduced absorption of selected nutrients, increased gastric microbiota and small intestine bacterial overgrowth, reduced immune response, tubular‐interstitial inflammation, increased bone turnover, and accumulation of amyloid in the brain.^27^

PPI use was also significantly associated with renal insufficiency even after adjusting for acute interstitial nephritis (AIN) in the Xie et al, 2019 VA cohort analysis. AIN is a drug reaction known to be caused by PPI.^28^ SRs of observational studies have found PPIs to be associated with chronic kidney disease (CKD).^29^ The finding of continued renal insufficiency even after adjustment suggested the existence of unrecognized AKI or chronic latent renal injury.^18^

## 4 DISCUSSION

An evidence‐based approach to interpretation of clinical trial data turns first to the hierarchy of evidence. RCTs are higher on the hierarchy than observational studies because randomization provides powerful protection against known and unknown confounders that observational studies do not. Given that the COMPASS findings were from an RCT and found no increase in all‐cause mortality and the observational studies found an increase in all‐cause mortality with PPI use, the hierarchy of evidence points to the interpretation that the RCT findings should be accepted and the observational findings understood as being most likely explained by an unidentified confounder.^30^

Pharmacovigilance—“the science and activities relating to the detection, assessment, understanding, and prevention of adverse effects or any other drug‐related problem”^31^—challenges the use of the hierarchy of evidence for evaluating drug risk:

[N]one of the methods … (experimental data, clinical trials, spontaneous notifications, case–control studies, cohort studies and data mining) should be considered as definitive for evaluating drug risk. It is only the **convergence of proofs** which allows final conclusions and decisions in pharmacovigilance. Thus, the notion of ‘levels of evidence’, widely used for evaluating drug efficacy, cannot be applied in the field of [Adverse Drug Reactions] ADRs; all methods are of interest for evaluation of ADRs.^32^

Insisting on RCT evidence for fatal and serious adverse events from medication use in real‐life populations contravenes modern standards in pharmacovigilance that are more directly applicable to the evaluation of the serious adverse events associated with medications.

Ethical constraints on designing RCTs to investigate the harms associated with drugs have driven innovation in observational study design. Studies like Xie et al, 2019 replicate the safety features of RCTs including comparable selection criteria for inclusion in the cohort, exposure definitions, covariate choices, outcome definitions, and analytic strategies.^33^ Older observational studies that use datasets to look for associations between the independent and dependent variables using factorial analyses are primitive by comparison. Clinicians are correct in being skeptical of associations that are in the range of OR and HR less than 2, given the vulnerability of such analyses to unrecognized confounders. In evaluating clinical data, analyses have “found little evidence that estimates of treatment effects in observational studies reported after 1984 are either consistently larger than or qualitatively different from those obtained in RCTs.” ^34^ The difficulties of capturing the harms of pharmaceutical use under routine clinical practice conditions are recognized to be even more difficult to capture under the “ideal” conditions of the RCT.^35^ Contemporary observational studies using the administrative datasets of large integrated health‐care systems provide advantages over RCTs of investigating the rate of serious adverse events.

To identify and control for unknown confounders, Xie et al, in an earlier 2017 study controlled for known risk factors including age, race, gender, estimated glomerular filtration rate (eGFR), number of serum creatinine measurements, number of hospitalizations, diabetes mellitus, hypertension, cardiovascular disease, peripheral artery disease, cerebrovascular disease, chronic lung disease, hepatitis C, HIV, dementia, cancer, gastroesophageal reflex disease, upper GI tract bleeding, ulcer disease, *H pylori* infection, Barrett's esophagus, achalasia, stricture, and esophageal adenocarcinoma. Then they tested for an uncontrolled confounder that would explain the finding of increased mortality using a rule‐out and external adjustment approach.^36^ They determined that a confounder would have to be twice as likely in PPI users (OR 2.0) and the HR of death associated with this uncontrolled confounder exceed 4.0 to explain their finding of excess mortality with PPI use. They concluded:

Given that our analyses accounted for most known strong independent risk factors of death and employed an active comparator group, to cancel the results, any uncontrolled confounder of the required prevalence (OR 2 or more …) and strength (HR 4 or more …) would also have to be independent of the confounders already adjusted for and is unlikely to exist; thus, the results cannot be fully explained by this putative uncontrolled confounder ^37^(p.6).

Additional features like propensity score analysis and using physician preferences as a calibration check on the analysis also provide important safeguards.

**Figure 2:**
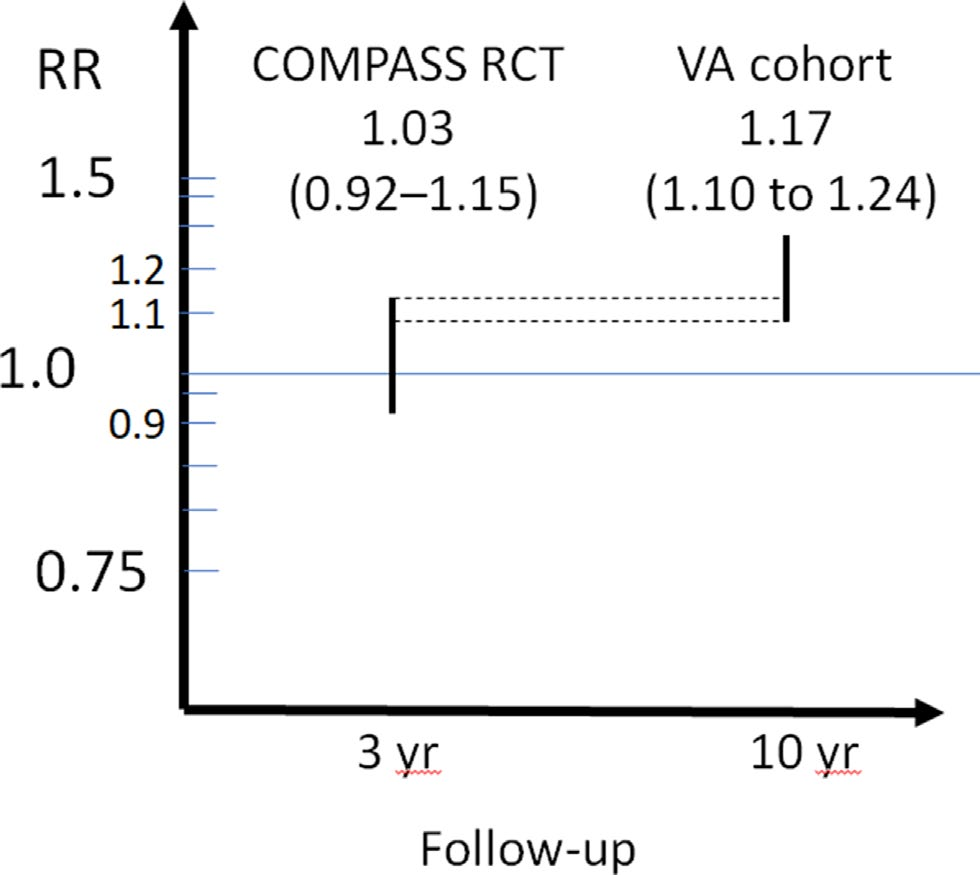
PPIs: All‐cause mortality—COMPASS trial vs VA cohort results

The 95% CI provides more accurate representation of reality than single point estimate. COMPASS researchers interpret their findings to “suggest PPI therapy is safe for up to a median of 3 years.^13^” They report being reassured that the HRs and ORs from their study “are lower than the lower end of the 95% CI” reported for all‐cause mortality in the Xie et al, 2017 initial analysis.^37^ However, the Xie et al, 2019 VA cohort study findings are not inconsistent with the COMPASS trial findings.^18^ There is an overlap in the 95% confidence intervals between VA cohort (1.10 to 1.24) and COMPASS trial (0.92‐1.15). The upper bound of the COMPASS trial 95% confidence interval virtually equals the point estimate of the cohort study of 1.15 to 1.17 (Figure 2). Thus, the data among mortality studies are not discordant but rather convergent. The results also show that the longer the duration of exposure to PPI, the greater the risk of death. There was a graded relation between duration of exposure and risks of all‐cause mortality, death due to cardiovascular diseases, cancers, and kidney diseases.^18^ This suggests that had the COMPASS RCT continued through to 10 years of follow‐up the confidence interval would have approached the VA cohort findings. Duration of use and study follow‐up could explain the seeming discordant findings.

In summary, careful appraisal of the totality of available evidence suggests that long‐term PPI utilization likely increases all‐cause mortality. The VA study reported that the longer the duration of exposure to PPI, the greater the risk of death. The findings presented here are limited to male, Caucasian with limited generalizability. It may not apply to cohorts with other characteristics because the VA and COMPASS datasets were predominately male (96%, 78%), Caucasian (87%, 60%), with a high average age range from 53 to 77 and 59 to 76 years, respectively. Generalizability may not extend to PPI users over 75, women, and other ethnic groups that are not without comparable overuse.

A prospective independent RCT is needed in older populations with multiple medical conditions with PPI durations of 3‐10 years inclusive of women and non‐Caucasians. In the meantime, promoting awareness of the potential to increase risk of death and other serious complications seems important. Assuming that these signals are artifacts is less responsible. We argue that it would be difficult to conduct a RCT of an adverse drug event on the scale of the included observational studies like VA cohort (A total of 214,467 included patients in VA vs 17,598 in COMPASS trial), an average exposure time ranges from 3 years to 4.6 years, with the length of follow‐up of 3 years compared to 10 years, respectively.

## 5 CONCLUSIONS AND IMPLICATIONS FOR PRACTICE

Our interpretive framework supports the principle that no one study or pooled analysis of studies can adequately determine whether the harm associated with drug therapy is real. A convergence of proof using data from various sources and study designs is needed. Considering the data from the COMPASS RCT together with the pharmaco‐epidemiology observational studies leads us to conclude that on balance, it is likely that long‐term PPI use increases all‐cause mortality in older adults. Given the high prevalence of long‐term PPI utilization, this message needs to be conveyed to health professionals and patients.

## Disclosures

None declared.

## Author Contributions

The authors are a group of Clinician‐scientists (Ben‐Eltriki, Green, Musini, Bassett and Wright), medical researcher and epidemiologist (Maclure) as well as Cochrane Hypertension authors and reviewers (Musini, Bassett and Wright). The authors are experts in analyzing clinical trials of drugs, and clarifying the state of scientific evidence regarding effectiveness and safety of drug therapy. All the authors participated in the study design, interpreted the data analysis, assessed the certainty of evidence, and revised the manuscript. Mohamed Ben‐Eltriki and Carolyn Green screened studies for eligibility, performed data extraction, assessed the risk of bias, performed data analysis, and wrote the first draft of the manuscript.

## Supporting information

Supplementary Material

## Data Availability

The data that supports the findings of this study are available in the supplementary material of this article. Supplementary file shows a bibliography sorted by harm type.
