# Supplementary material for: Do proton pump inhibitors increase mortality? A systematic review and in‐depth analysis of the evidence
Source: Pharmacol Res Perspect. 2020 Sep 30;8(5):e00651. doi: 10.1002/prp2.651 (PMC7525804; doi:10.1002/prp2.651)
Supplement: Supplementary file 1 — Supplementary Material [file PRP2-8-e00651-s001.docx]

| 103 recent systematic reviews of specific harms  associated with Proton Pump InhibitorsInfectionsClostridium Difficile  1. Cao F, Chen CX, Wang M, Liao HR, Wang MX, Hua SZ, et al. Updated meta-analysis of controlled observational studies: proton-pump inhibitors and risk of Clostridium difficile infection. J Hosp Infect. 2018;98(1):4-13. 2. Azab M, Doo L, Doo DH, Elmofti Y, Ahmed M, Cadavona JJ, et al. Comparison of the Hospital-Acquired Clostridium difficile Infection Risk of Using Proton Pump Inhibitors versus Histamine-2 Receptor Antagonists for Prophylaxis and Treatment of Stress Ulcers: A Systematic Review and Meta-Analysis. Gut and liver. 2017;11(6):781-8. 3. Miao-Miao Z, Hui-Lan L, Jia L, Cong L, Zan-Ling Z. Association between use of proton pump inhibitors and the risk of clostridium difficile infection: A meta-analysis. Chinese Journal of Evidence-Based Medicine. 2016;16(3):278-85. 4. Oshima T, Wu L, Li M, Fukui H, Watari J, Miwa H. Magnitude and direction of the association between Clostridium difficile infection and proton pump inhibitors in adults and pediatric patients: a systematic review and meta-analysis. Journal of Gastroenterology. 2018;53(1):84-94. 5. Tariq R, Singh S, Gupta A, Pardi DS, Khanna S. Association of Gastric Acid Suppression With Recurrent Clostridium difficile Infection: A Systematic Review and Meta-analysis. JAMA Internal Medicine. 2017;177(6):784-91. 6. Furuya-Kanamori L, Stone JC, Clark J, McKenzie SJ, Yakob L, Paterson DL, et al. Comorbidities, Exposure to Medications, and the Risk of Community-Acquired Clostridium difficile Infection: a systematic review and meta-analysis. Infect Control Hosp Epidemiol. 2015;36(2):132-41. 7. Deshpande A, Pasupuleti V, Thota P, Pant C, Rolston DD, Hernandez AV, et al. Risk factors for recurrent Clostridium difficile infection: a systematic review and meta-analysis. Infect Control Hosp Epidemiol. 2015;36(4):452-60. 8. Arriola V, Tischendorf J, Musuuza J, Barker A, Rozelle JW, Safdar N. Assessing the Risk of Hospital-Acquired Clostridium Difficile Infection With Proton Pump Inhibitor Use: A Meta-Analysis. Infect Control Hosp Epidemiol. 2016;37(12):1408-17. 9. Avendano-Reyes JM. Association between proton pump inhibitors therapy and Clostridium diffcile infection: Myth or fact?. [Spanish]. Medicina Interna de Mexico. 2016;32(5):561-8.  Bacterial peritonitis  1. Yu T, Tang Y, Jiang L, Zheng Y, Xiong W, Lin L. Proton pump inhibitor therapy and its association with spontaneous bacterial peritonitis incidence and mortality: A meta-analysis. Dig Liver Dis. 2016;48(4):353-9. 2. Zhong HJ, Lin D, Lu ZY, Yang WY, Chen Y. Use of gastric-acid suppressants may be a risk factor for enteric peritonitis in patients undergoing peritoneal dialysis: A meta-analysis. J Clin Pharm Ther. 2018;17:17. 3. Khan MA, Kamal S, Khan S, Lee WM, Howden CW. Systematic review and meta-analysis of the possible association between pharmacological gastric acid suppression and spontaneous bacterial peritonitis. Eur J Gastroenterol Hepatol. 2015;27(11):1327-36. 4. Yu T, Lin L. Proton pump inhibitor therapy and its association with spontaneous bacterial peritonitis incidence and mortality: A meta-analysis. Journal of Digestive Diseases. 2015:95-6.  Hepatic Encephalopathy  1. Bian J, Wang A, Lin J, Wu L, Huang H, Wang S, et al. Association between proton pump inhibitors and hepatic encephalopathy: A meta-analysis. Medicine (Baltimore). 2017;96(17):e6723.  Community-acquired Enteric Infection  1. Hafiz RA, Wong C, Paynter S, David M, Peeters G. The Risk of Community-Acquired Enteric Infection in Proton Pump Inhibitor Therapy: Systematic Review and Meta-analysis. Annals of Pharmacotherapy. 2018;52(7):613-22.  Community acquired pneumoniaLambert AA, Lam JO, Paik JJ, Ugarte-Gil C, Drummond MB, Crowell TA. Risk of community-acquired pneumonia with outpatient proton-pump inhibitor therapy: a systematic review and meta-analysis. PLoS ONE. 2015;10(6):e0128004.  1. Wang CH, Li CH, Hsieh R, Fan CY, Hsu TC, Chang WC, et al. Proton pump inhibitors therapy and the risk of pneumonia: a systematic review and meta-analysis of randomized controlled trials and observational studies. Expert Opin Drug Saf. 2019;18(3):163-72.  Gut Dysbiosis  1. Le Bastard Q, Al-Ghalith GA, Gregoire M, Chapelet G, Javaudin F, Dailly E, et al. Systematic review: human gut dysbiosis induced by non-antibiotic prescription medications. Aliment Pharmacol Ther. 2018;47(3):332-45. 2. Naito Y, Kashiwagi K, Takagi T, Andoh A, Inoue R. Intestinal Dysbiosis Secondary to Proton-Pump Inhibitor Use. Digestion. 2018;97(2):195-204. 3. Li Z, Wu C, Li L, Wang Z, Xie H, He X, et al. Effect of long-term proton pump inhibitor administration on gastric mucosal atrophy: A meta-analysis. Saudi j. 2017;23(4):222-8.  Small intestinal bacterial overgrowth  1. Su T, Lai S, Lee A, He X, Chen S. Meta-analysis: proton pump inhibitors moderately increase the risk of small intestinal bacterial overgrowth. Journal of Gastroenterology. 2018;53(1):27-36. 2. Chen B, Kim JJ, Zhang Y, Du L, Dai N. Prevalence and predictors of small intestinal bacterial overgrowth in irritable bowel syndrome: a systematic review and meta-analysis. Journal of Gastroenterology. 2018;14:14. 3. Su T, Lai S, Lee A, He X, Chen S. Meta-analysis: proton pump inhibitors moderately increase the risk of small intestinal bacterial overgrowth. Journal of Gastroenterology. 2018;53(1):27-36. 4. Chen B, Kim JJ, Zhang Y, Du L, Dai N. Prevalence and predictors of small intestinal bacterial overgrowth in irritable bowel syndrome: a systematic review and meta-analysis. Journal of Gastroenterology. 2018;14:14.  Microscopic colitis  1. Zarinafsar S, Matine L, Razzak E, Li K, Kim E, Yang S, et al. Does long-term use of a proton pump inhibitor (PPI) lead to osteopenia or fractures? Journal of Investigative Medicine. 2018;66 (1):168-9.  \| Bone Fractures / Falls  1. Poly TN, Islam MM, Yang HC, Wu CC, Li YJ. Proton pump inhibitors and risk of hip fracture: a meta-analysis of observational studies. Osteoporos Int. 2019;30(1):103-14. 2. Liu J, Li X, Fan L, Yang J, Wang J, Sun J, et al. Proton pump inhibitors therapy and risk of bone diseases: An update meta-analysis. Life Sci. 2019;218:213-23. 3. Lapumnuaypol K, Thongprayoon C, Wijarnpreecha K, Tiu A, Cheungpasitporn W. Risk of Fall in Patients Taking Proton Pump Inhibitors: A Meta-Analysis. QJM : monthly journal of the Association of Physicians. 2019;112(2):115-21. 4. Fan XD, Ayom MIN, Sun WG, Yin PP, Wang XY, Jia A, et al. An updated meta-analysis: The effect of proton pump inhibitor on risk of osteoporosis and fracture. International Journal of Clinical and Experimental Medicine. 2017;10(11):15680-95. 5. Dubcenco E, Beers-Block PM, Kim LP, Schotland P, Levine JG, McCloskey CA, et al. A Proton Pump Inhibitor in the Reformulation Setting: Bioequivalence and Potential Implications for Long-Term Safety. Clinical and Translational Science. 2017;10(5):387-94. 6. Hussain S, Siddiqui AN, Habib A, Hussain MS, Najmi AK. Proton pump inhibitors' use and risk of hip fracture: a systematic review and meta-analysis. Rheumatology international. 2018;38(11):1999-2014. 7. Nassar Y, Richter S. Proton-pump Inhibitor Use and Fracture Risk: An Updated Systematic Review and Meta-analysis. J. 2018;25(3):141-51. 8. Yang SD, Chen Q, Wei HK, Zhang F, Yang DL, Shen Y, et al. Bone fracture and the interaction between bisphosphonates and proton pump inhibitors: a meta-analysis. International journal of clinical and experimental medicine. 2015;8(4):4899-910. 9. Cai D, Feng W, Jiang Q. Acid-suppressive medications and risk of fracture: An updated meta-analysis. International Journal of Clinical and Experimental Medicine. 2015;8(6):8893-904.  Kidney Disease  1. Sun J, Sun H, Cui M, Sun Z, Li W, Wei J, et al. The use of anti-ulcer agents and the risk of chronic kidney disease: a meta-analysis. Int Urol Nephrol. 2018;50(10):1835-43. 2. Wu B, Shang W, Li Y, Ren Y, Liu Z, Wei H, et al. Association between proton pump inhibitors use and kidney diseases: A meta-analysis. International Journal of Clinical and Experimental Medicine. 2018;11(7):6465-73. 3. Hussain S, Singh A, Habib A, Najmi AK. Proton pump inhibitors use and risk of chronic kidney disease: Evidence-based meta-analysis of observational studies. Clinical Epidemiology and Global Health. 2019;7(1):46-52. 4. Nochaiwong S, Ruengorn C, Awiphan R, Koyratkoson K, Chaisai C, Noppakun K, et al. The association between proton pump inhibitor use and the risk of adverse kidney outcomes: a systematic review and meta-analysis. Nephrology Dialysis Transplantation. 2018;33(2):331-42. 5. Qiu T, Zhou J, Zhang C. Acid-suppressive drugs and risk of kidney disease: A systematic review and meta-analysis. J Gastroenterol Hepatol. 2018;12:12. 6. Hussain S, Singh A, Habib A, Najmi AK. Proton pump inhibitors use and the risk of chronic kidney disease: A systematic review and meta analysis. Nephrology Dialysis Transplantation. 2017:iii553-554. 7. Wu B, Shang W, Li Y, Ren Y, Liu Z, Wei H, et al. Association between proton pump inhibitors use and kidney diseases: A meta-analysis. International Journal of Clinical and Experimental Medicine. 2018;11(7):6465-73.  CancerGastric Cancer  1. Wan QY, Wu XT, Li N, Du L, Zhou Y. Long-term proton pump inhibitors use and risk of gastric cancer: a meta-analysis of 926 386 participants. Gut. 2018;03:03.  Hepatocellular carcinoma  1. Zhao J, Hua L, Li N, An R, Liang C. Letter: proton pump inhibitors use and risk of hepatocellular carcinoma: A meta-analysis of observational studies. Alimentary Pharmacology and Therapeutics. 2018;48(8):886-8.  Esophageal Adenocarcinoma In Barrett’s Esophagus  1. Hu Q, Sun TT, Hong J, Fang JY, Xiong H, Meltzer SJ. Proton Pump Inhibitors Do Not Reduce the Risk of in Patients with Barrett's Esophagus: A Systematic Review and Meta-Analysis. PLoS ONE. 2017;12(1):e0169691.  Fundic gland polyps  1. Martin FC, Chenevix-Trench G, Yeomans ND. Systematic review with meta-analysis: fundic gland polyps and proton pump inhibitors. Aliment Pharmacol Ther. 2016;44(9):915-25.  Cardiovascular Events  1. Shiraev TP, Bullen A. Proton Pump Inhibitors and Cardiovascular Events: A Systematic Review. Heart Lung Circ. 2018;27(4):443-50. 2. Sun S, Cui Z, Zhou M, Li R, Li H, Zhang S, et al. Proton pump inhibitor monotherapy and the risk of cardiovascular events in patients with gastro-esophageal reflux disease: a meta-analysis. Neurogastroenterol Motil. 2017;29(2). 3. Al-Shammari M, Shah S, Maklad M, Yoo JW, Makar RS. Do proton pump inhibitors really increase cardiovascular risk? A systematic review and meta-analyses of existing literature. American Journal of Gastroenterology. 2017;112 (Supplement 1):S666-S7.  Dementia  1. Li M, Luo Z, Yu S, Tang Z. Proton pump inhibitor use and risk of dementia: Systematic review and meta-analysis. Medicine (Baltimore). 2019;98(7):e14422.  Batchelor R, Gilmartin JF, Kemp W, Hopper I, Liew D. Dementia, cognitive impairment and proton pump inhibitor therapy: A systematic review. J Gastroenterol Hepatol. 2017;32(8):1426-35.Drug Drug Interactions  1. Shamliyan TA, Middleton M, Borst C. Patient-centered Outcomes with Concomitant Use of Proton Pump Inhibitors and Other Drugs. Clinical Therapeutics. 2017;39(2):404-27.e36. 2. Yucel E, Sancar M, Yucel A, Okuyan B. Adverse drug reactions due to drug-drug interactions with proton pump inhibitors: assessment of systematic reviews with AMSTAR method. Expert Opinion on Drug Safety. 2016;15(2):223-36.  Dental implant failure  1. Chappuis V, Avila-Ortiz G, Araujo MG, Monje A. Medication-related dental implant failure: Systematic review and meta-analysis. Clin Oral Implants Res. 2018;29 Suppl 16:55-68.  Myopathy  1. Colmenares EW, Pappas AL. Proton Pump Inhibitors: Risk for Myopathy? Annals of Pharmacotherapy. 2017;51(1):66-71.  LT use (over 3 months)  1. Islam MM, Poly TN, Walther BA, Dubey NK, Anggraini Ningrum DN, Shabbir SA, et al. Adverse outcomes of long-term use of proton pump inhibitors: a systematic review and meta-analysis. Eur J Gastroenterol Hepatol. 2018;30(12):1395-405.  Childhood asthma following use in pregnancy  1. Lai T, Wu M, Liu J, Luo M, He L, Wang X, et al. Acid-Suppressive Drug Use During Pregnancy and the Risk of Childhood Asthma: A Meta-analysis. Pediatrics. 2018;141(2).  Laboratory findingsGlycemic Control  1. Gomez-Izquierdo JC, Yu OHY. The Influence of Proton-Pump Inhibitors on Glycemic Control: A Systematic Review of the Literature and a Meta-Analysis. Can. 2017;41(4):351-61.  Gastrin Levels and Gastric Histology  1. Lundell L, Vieth M, Gibson F, Nagy P, Kahrilas PJ. Systematic review: the effects of long-term proton pump inhibitor use on serum gastrin levels and gastric histology. Aliment Pharmacol Ther. 2015;42(6):649-63.  Hypomagnesemia  1. Janett S, Camozzi P, Peeters GGAM, Lava SAG, Simonetti GD, Simonetti BG, et al. Hypomagnesemia Induced by Long-Term Treatment with Proton-Pump Inhibitors. Gastroenterology Research and Practice. 2015;2015 (no pagination)(951768). 2. Cheungpasitporn W, Thongprayoon C, Kittanamongkolchai W, Srivali N, Edmonds PJ, Ungprasert P, et al. Proton pump inhibitors linked to hypomagnesemia: a systematic review and meta-analysis of observational studies. Ren Fail. 2015;37(7):1237-41. 3. Park CH, Kim EH, Roh YH, Kim HY, Lee SK. The association between the use of proton pump inhibitors and the risk of hypomagnesemia: a systematic review and meta-analysis. PLoS ONE. 2014;9(11):e112558.  Hospitalization  1. Wang KN, Bell JS, Chen EYH, Gilmartin-Thomas JFM, Ilomaki J. Medications and Prescribing Patterns as Factors Associated with Hospitalizations from Long-Term Care Facilities: A Systematic Review. Drugs Aging. 2018;35(5):423-57.  AE data from reviews of Tx comparisonsTx H Pylori  1. Dong SQ, Singh TP, Wei X, Yao H, Wang HL. Review: A Japanese population-based meta-analysis of vonoprazan versus PPI for Helicobacter pylori eradication therapy: Is superiority an illusion? Helicobacter. 2017;22(6). 2. Nyssen OP, McNicholl AG, Megraud F, Savarino V, Oderda G, Fallone CA, et al. Sequential versus standard triple first-line therapy for Helicobacter pylori eradication. Cochrane Database of Systematic Reviews. 2016(6):CD009034. 3. Zeng Y, Ye Y, Liang D, Guo C, Li L. Meta-analysis of the efficacy of lansoprazole and omeprazole for the treatment of H.pylori-associated duodenal ulcer. Int J Physiol Pathophysiol Pharmacol. 2015;7(3):158-64. 4. Wang B, Wang YH, Lv ZF, Xiong HF, Wang H, Yang Y, et al. Review: efficacy and safety of hybrid therapy for Helicobacter pylori infection: a systematic review and meta-analysis. Helicobacter. 2015;20(2):79-88. 5. Ford AC, Gurusamy SK, Delaney B, Forman D, Moayyedi P. Eradication therapy for peptic ulcer disease in Helicobacter pylori-positive people. Cochrane Database of Systematic Reviews. 2016(4). 6. Liu X, Wang H, Lv Z, Wang Y, Wang B, Xie Y, et al. Rescue Therapy with a Proton Pump Inhibitor Plus Amoxicillin and Rifabutin for Helicobacter pylori Infection: A Systematic Review and Meta-Analysis. Gastroenterol Res Pract. 2015;2015:415648.  Tx Peptic Ulcer  1. Scally B, Emberson JR, Spata E, Reith C, Davies K, Halls H, et al. Effects of gastroprotectant drugs for the prevention and treatment of peptic ulcer disease and its complications: a meta-analysis of randomised trials. Lancet Gastroenterol Hepatol. 2018;3(4):231-41. 2. Jian Z, Li H, Race NS, Ma T, Jin H, Yin Z. Is the era of intravenous proton pump inhibitors coming to an end in patients with bleeding peptic ulcers? Meta-analysis of the published literature. British Journal of Clinical Pharmacology. 2016;82(3):880-9. 3. Gurusamy SK, Pallari E. Medical versus surgical treatment for refractory or recurrent peptic ulcer. Cochrane Database of Systematic Reviews. 2016(3).  Tx of Gastroesophageal Varices  1. Zhu J, Qi X, Yu H, Su C, Guo X. Acid suppression in patients treated with endoscopic therapy for the management of gastroesophageal varices: a systematic review and meta-analysis. Expert rev. 2018;12(6):617-24. 2. Lo EA, Wilby KJ, Ensom MH. Use of proton pump inhibitors in the management of gastroesophageal varices: a systematic review. Annals of Pharmacotherapy. 2015;49(2):207-19.  Tx GI Bleeding  1. Szabo IL, Matics R, Hegyi P, Garami A, Illes A, Sarlos P, et al. PPIs Prevent Aspirin-Induced Gastrointestinal Bleeding Better than H2RAs. A Systematic Review and Meta-analysis. J. 2017;26(4):395-402.  Tx Upper GI Bleeding After Endoscopy  1. Zhang YS, Li Q, He BS, Liu R, Li ZJ. Proton pump inhibitors therapy vs H2 receptor antagonists therapy for upper gastrointestinal bleeding after endoscopy: A meta-analysis. World Journal of Gastroenterology. 2015;21(20):6341-51.  Tx of GERD  1. Mei J, Yu Y, Ma J, Yu X. Evaluation of the effectiveness of esomeprazole treatment strategies in the management of patients with gastroesophageal reflux disease symptoms: a meta-analysis. Pharmazie. 2016;71(5):285-91.  Tx of GERD in Idiopathic Pulmonary Fibrosis  1. Fidler L, Sitzer N, Shapera S, Shah PS. Treatment of Gastroesophageal Reflux in Patients With Idiopathic Pulmonary Fibrosis: A Systematic Review and Meta-Analysis. Chest. 2018;153(6):1405-15.  Tx Chronic Laryngitis  1. Yang Y, Wu H, Zhou J. Efficacy of acid suppression therapy in gastroesophageal reflux disease-related chronic laryngitis. Medicine (Baltimore). 2016;95(40):e4868.  Tx Preventing NSAID-associated gastrointestinal toxicity  1. Yuan JQ, Tsoi KK, Yang M, Wang JY, Threapleton DE, Yang ZY, et al. Systematic review with network meta-analysis: comparative effectiveness and safety of strategies for preventing NSAID-associated gastrointestinal toxicity. Aliment Pharmacol Ther. 2016;43(12):1262-75.  Preventive Tx in Critically Ill Patients / ICU/ Neurocritical care  1. Toews I, George AT, Peter JV, Kirubakaran R, Fontes LES, Ezekiel JPB, et al. Interventions for preventing upper gastrointestinal bleeding in people admitted to intensive care units. Cochrane Database of Systematic Reviews. 2018;6:CD008687. 2. Sridharan K, Sivaramakrishnan G, Gnanaraj J. Pharmacological interventions for stress ulcer prophylaxis in critically ill patients: a mixed treatment comparison network meta-analysis and a recursive cumulative meta-analysis. Expert Opinion on Pharmacotherapy. 2018;19(2):151-8. 3. Barbateskovic M, Marker S, Granholm A, Anthon CT, Krag M, Jakobsen JC, et al. Stress ulcer prophylaxis with proton pump inhibitors or histamin-2 receptor antagonists in adult intensive care patients: a systematic review with meta-analysis and trial sequential analysis. Intensive Care Medicine. 2019;45(2):143-58. 4. Reynolds PM, MacLaren R. Re-evaluating the Utility of Stress Ulcer Prophylaxis in the Critically Ill Patient: A Clinical Scenario-Based Meta-Analysis. Pharmacotherapy. 2018;13:13. 5. Sridharan K, Sivaramakrishnan G, Gnanaraj J. Pharmacological interventions for stress ulcer prophylaxis in critically ill patients: a mixed treatment comparison network meta-analysis and a recursive cumulative meta-analysis. Expert Opinion on Pharmacotherapy. 2018;19(2):151-8. 6. Toews I, George AT, Peter JV, Kirubakaran R, Fontes LES, Ezekiel JPB, et al. Interventions for preventing upper gastrointestinal bleeding in people admitted to intensive care units. Cochrane Database of Systematic Reviews. 2018;6:CD008687. 7. Liu B, Liu S, Yin A, Siddiqi J. Risks and benefits of stress ulcer prophylaxis in adult neurocritical care patients: a systematic review and meta-analysis of randomized controlled trials. Crit Care. 2015;19:409. 8. Alshamsi F, Belley-Cote E, Cook D, Almenawer SA, Alqahtani Z, Perri D, et al. Efficacy and safety of proton pump inhibitors for stress ulcer prophylaxis in critically ill patients: a systematic review and meta-analysis of randomized trials. Crit Care. 2016;20(1):120. 9. Barletta JF, Bruno JJ, Buckley MS, Cook DJ. Stress Ulcer Prophylaxis. Critical Care Medicine. 2016;44(7):1395-405. 10. Alhazzani W, Alshamsi F, Belley-Cote E, Heels-Ansdell D, Brignardello-Petersen R, Alquraini M, et al. Efficacy and safety of stress ulcer prophylaxis in critically ill patients: a network meta-analysis of randomized trials. Intensive Care Medicine. 2018;44(1):1-11.  Preventive Tx Cardiac/ angioplasty/ concomitant use with dual antiplatelet therapy/ clopidogrel/ aspirin  1. Khan SU, Lone AN, Asad ZUA, Rahman H, Khan MS, Saleem MA, et al. Meta-analysis of efficacy and safety of proton pump inhibitors with dual antiplatelet therapy for coronary artery disease. Cardiovasc Revasc Med. 2019;10:10. 2. Almufleh A, Ramirez FD, So D, Le May M, Chong AY, Torabi N, et al. H2 Receptor Antagonists versus Proton Pump Inhibitors in Patients on Dual Antiplatelet Therapy for Coronary Artery Disease: A Systematic Review. Cardiology. 2018;140(2):115-23. 3. Bundhun PK, Teeluck AR, Bhurtu A, Huang WQ. Is the concomitant use of clopidogrel and Proton Pump Inhibitors still associated with increased adverse cardiovascular outcomes following coronary angioplasty?: a systematic review and meta-analysis of recently published studies (2012 - 2016). BMC Cardiovasc Disord. 2017;17(1):3. 4. Dahal K, Sharma SP, Kaur J, Anderson BJ, Singh G. Efficacy and Safety of Proton Pump Inhibitors in the Long-Term Aspirin Users: A Meta-Analysis of Randomized Controlled Trials. American Journal of Therapeutics. 2017;24(5):e559-e69. 5. Demcsak A, Lantos T, Balint ER, Hartmann P, Vincze A, Bajor J, et al. PPIs Are Not Responsible for Elevating Cardiovascular Risk in Patients on Clopidogrel-A Systematic Review and Meta-Analysis. Front Physiol. 2018;9:1550. 6. Hu W, Tong J, Kuang X, Chen W, Liu Z. Influence of proton pump inhibitors on clinical outcomes in coronary heart disease patients receiving aspirin and clopidogrel: A meta-analysis. Medicine (Baltimore). 2018;97(3):e9638. 7. Khan MY, Siddiqui WJ, Alvarez C, Aggarwal S, Hasni SF, Ahmad A, et al. Reduction in postpercutaneous coronary intervention angina in addition to gastrointestinal events in patients on combined proton pump inhibitors and dual antiplatelet therapy: a systematic review and meta-analysis. Eur J Gastroenterol Hepatol. 2018;30(8):847-53. 8. Niu Q, Wang Z, Zhang Y, Wang J, Zhang P, Wang C, et al. Combination Use of Clopidogrel and Proton Pump Inhibitors Increases Major Adverse Cardiovascular Events in Patients With Coronary Artery Disease: A Meta-Analysis. J Cardiovasc Pharmacol Ther. 2017;22(2):142-52. 9. Malhotra K, Katsanos AH, Bilal M, Ishfaq MF, Goyal N, Tsivgoulis G. Cerebrovascular Outcomes With Proton Pump Inhibitors and Thienopyridines: A Systematic Review and Meta-Analysis. Stroke. 2018;49(2):312-8. 10. Li JX, Jin EZ, Li Y, Song ZY, Liu SH, Yan SJ, et al. Lack increased evidence of cardiovascular events in patients receiving clopidogrel with proton-pump inhibitors: A meta-analysis and system review. International Journal of Clinical and Experimental Medicine. 2018;11(11):11481-93. 11. Serbin MA, Guzauskas GF, Veenstra DL. Clopidogrel-Proton Pump Inhibitor Drug-Drug Interaction and Risk of Adverse Clinical Outcomes Among PCI-Treated ACS Patients: A Meta-analysis. J Manag Care Spec Pharm. 2016;22(8):939-47. 12. Yi ZM, Qiu TT, Zhang Y, Liu ZY, Zhai SD. Comparison of prophylactic effect of UGIB and effects on platelet function between PPIs and H<sub>2</sub>RAs combined with DAPT: systematic review and meta-analysis. Ther Clin Risk Manag. 2017;13:367-77. 13. Wang ZY, Chen M, Zhu LL, Yu LS, Zeng S, Xiang MX, et al. Pharmacokinetic drug interactions with clopidogrel: updated review and risk management in combination therapy. Ther Clin Risk Manag. 2015;11:449-67. \| \| --- \|  1. Law EH, Badowski M, Hung YT, Weems K, Sanchez A, Lee TA. Association Between Proton Pump Inhibitors and Microscopic Colitis. Annals of Pharmacotherapy. 2017;51(3):253-63 |
| --- | --- |
